# Experiencing sweet taste is associated with an increase in prosocial behavior

**DOI:** 10.1038/s41598-023-28553-9

**Published:** 2023-02-02

**Authors:** Michael Schaefer, Anja Kühnel, Felix Schweitzer, Franziska Rumpel, Matti Gärtner

**Affiliations:** 1 Medical School Berlin, Calandrellistr. 1-9, 12247 Berlin, Germany; 2 Otto-von-Guericke Business School Magdeburg, 39106 Magdeburg, Germany

**Keywords:** Cognitive neuroscience, Social behaviour, Social neuroscience

## Abstract

Taste may be the first sense that emerged in evolution. Taste is also a very important sense since it signals potential beneficial or dangerous effects of foods. Given this fundamental role of taste in our lives, it is not surprising that taste also affects our psychological perception and thinking. For example, previous research demonstrated remarkable psychological effects of sweet taste experiences, suggesting that sweetness may be a source domain for prosocial functioning. Recent research reports that briefly experiencing sweet taste made participants more helpful in their intentions and behavior. The current study aims to test this hypothesis and to examine the neural underpinnings of this effect by using an fMRI approach. Participants were asked to taste sweet, salty, and neutral taste while lying in the fMRI scanner. Subsequently their prosocial behavior was tested by playing the dictator game, a measure of prosocial behavior. Results showed that sweet taste was associated with an increase in prosocial behavior compared with previously experiencing salty taste but did not affect control stimuli ratings. FMRI results revealed a modulation of the dorsal anterior cingulate cortex associated with this sweetness effect. This brain area is known to play a central role for monitoring conflicts and decisions and has been directly linked to selfish and prosocial economic decisions. The results demonstrate that sweet taste has complex psychological effects including positive and socially desirable outcomes. We discuss the results with other studies on psychological sweetness effects and suggest possible implications of these findings.

## Introduction

When we perceive the world around us, we can only do so with our bodies. Thus, our body can be seen as an interface between our cognitions and our environment. Theories and studies linked to the term embodiment have tried to describe the role of our body in these complex relationships, arguing that our cognitions are not independent from our senses^1,2^. In this view, our thoughts, impressions, judgements, or feelings can be influenced (or grounded) by basic sensory perception experiences, sometimes more than we may want to admit to ourselves^3,4^.

One example is taste. From an evolutionary point of view taste is one of our most important senses. Bitter taste provides information about potential toxic plants, whereas sweet taste (which preference may be innate to all of us) provides valuable information for detecting plants with available glucose^5,6^. There are also remarkable studies demonstrating links of taste with our cognitions. For example, previous research showed that the experience of physical disgust (a bitter taste) induced feelings of moral disgust, especially in participants with politically conservative attitudes^7^. Furthermore, similar facial motor activity for taste disgust, disgust in moral situations, and when responding to disgusting pictures have been found, suggesting overlapping neurophysiological mechanisms or “oral origins of moral disgust”^8^.

Beyond the link of bitter taste and moral disgust, there is also another taste, which potential psychological effects have been subject to recent research: sweetness. Sweet taste seems to be linked to romantic feelings and approaching attitudes. For example, Chan et al. reported that participants tasted plain distilled water as sweeter when they were asked beforehand to write about a time in which they have felt romantic love^9^. Similarly, Ren et al. showed that participants who previously consumed a sweet snack (as opposed to a salty snack or distilled water) rated a hypothetical relationship more positively than those who experienced a non-sweet snack and even made individuals more interested in starting a new romantic relationship^10^. Further studies report similar results on the link between sweetness and the activation of romantic mindsets and approaching attitudes^11–13^.

So why is sweetness so closely associated with romantic mindsets and approaching behavior? Many researchers explain this link with the conceptual or embodied metaphor theory. According to this theory (at least some) metaphors are not mere figures of speech, but affect the way we perceive, think, and behave^14,15^. Those conceptual metaphors might be based on sensory or bodily experiences we once made in our early life, which now work as scaffolds in our present decisions, perceptions, and cognitive operations^16^. A very prominent and perhaps conceptual metaphor seems to be that love is sweet, which has been described in many languages (e.g., English, German, Mandarin) and which may drive the effects we described above^10^.

Beyond the link of sweetness with love this taste seems to have even further intriguing effects on our thinking and behavior. In a series of experiments, Meier et al. showed that briefly tasting something sweet resulted in higher prosocial intentions and behavior. For example, in one study the authors asked their students to participate in a “taste experiment”, in which they had to rate the taste of sweet or salty snacks (chocolate or salty cracker). During the experiment a professor stopped by and requested volunteers for another (unrelated) study. The participants were asked how many minutes they would be willing to help. The authors took the answers as a measure for prosocial intentions. Results revealed that the experience of sweet taste before made the participants decisions more social and more willing to help^17^. These results have been supported by further studies^11,18^. Again the results have been explained by the conceptual metaphor theory, since in the English language sweetness is also often used to describe a social and helpful individual (“being a sweetie”). However, since this metaphor is not known in the German language, at least some results cannot be explained by this metaphor. Thus, alternative explanations beyond language theories have to be discussed as well. For example, since breast/formula milk tastes sweet^19^, the relationship between helping behavior and sweet taste (and perhaps also with love) may be based on feeding behaviors during infancy. This could also have been resulted in a scaffold for our present thinking and behavior (but not necessarily linked to language). Thus, it remains unclear why and how the experience of sweetness makes ourselves social.

Consumption of sweet foods has been shown to be associated not only with activation in primary gustatory processing brain areas but also linked to reward processing (e.g.,^20^). Given that a relationship between reward and prosocial behavior is known^21,22^ and behavioral studies found that feeling grateful was linked to the consumption of sweets^23^, the prosocial effects of sweet taste experience might also be explained by an engagement of the reward system. However, behavioral data showed that thankfulness for receiving a snack did not explain the more social behavior subsequent the experience of sweet taste^11^. In addition, it seems unclear whether it is the (sweet) taste or the calories that provide the link to reward^24^. The experiments on the psychological sweet effect reported above showed effects immediately after tasting, making it unlikely that the sugar of the sweet snacks was already metabolized.

The present study aims to address the question how sweet taste affects social behavior. To understand the neural underpinnings of the sweetness effect, we investigated the effect by means of an fMRI study. Participants tried sweet, salty, or neutral probes and were subsequently asked to play the dictator game (DG), which is used as a measure for prosocial behavior. We hypothesized that experiencing sweet taste make the participants behave more social, as reported before. Furthermore, we assumed that the neural underpinnings of this effect include brain areas known to be related to social behavior and decisions, in particular the right temporoparietal junction area (rTPJ), the anterior cingulate cortex (ACC), the insula, and prefrontal cortex. The rTPJ represents theory-of-mind engagement and has been related to social behavior^25,26^. ACC, prefrontal cortex, and insula have often been reported to be related to empathy and social decision processes in general^27–30^.

Moreover, we hypothesized that the sweet taste effect engages brain areas involved in primary taste processing, which are known to include especially the insula (among other regions such as the opercular cortex, pre- and postcentral gyrus and globus pallidus,^31^). This hypothesis points to the assumption that the sweetness metaphor is embodied, as outlined above^3,4,14–16^. According to this theory, activating the metaphor sweetness should elicit source-domain activity in primary processing areas (e.g., the insula) when individuals decide to behave social (but not when deciding selfish or during control conditions) in a subsequent decision task^32^.

## Materials and methods

### Participants

22 individuals (16 females) with a mean age of 22.50 years (±2.89 standard deviation) participated in the study. Sample size was calculated on similar fMRI studies on conceptual metaphor theory and embodiment (e.g.,^33–35^, for an overview see^36^). Participants were right-handed native German volunteers and had no neurological or psychiatric history. All participants gave written informed consent. The study adhered to the Declaration of Helsinki and was ethically approved by the ethics committee of the Medical School Berlin (Germany).

### Procedure

Participants were told a cover story that they would perform two separate and independent experiments in the fMRI scanner. While the first experiment was described to examine neural representation of taste sensations, the second one was linked to an investigation of brain correlates of behavior in an economic decision game. Participants were naive to the real goal of our study and debriefed and probed for suspicions about the experiment’s true aim at the end of the experiment.

**Figure 1:**
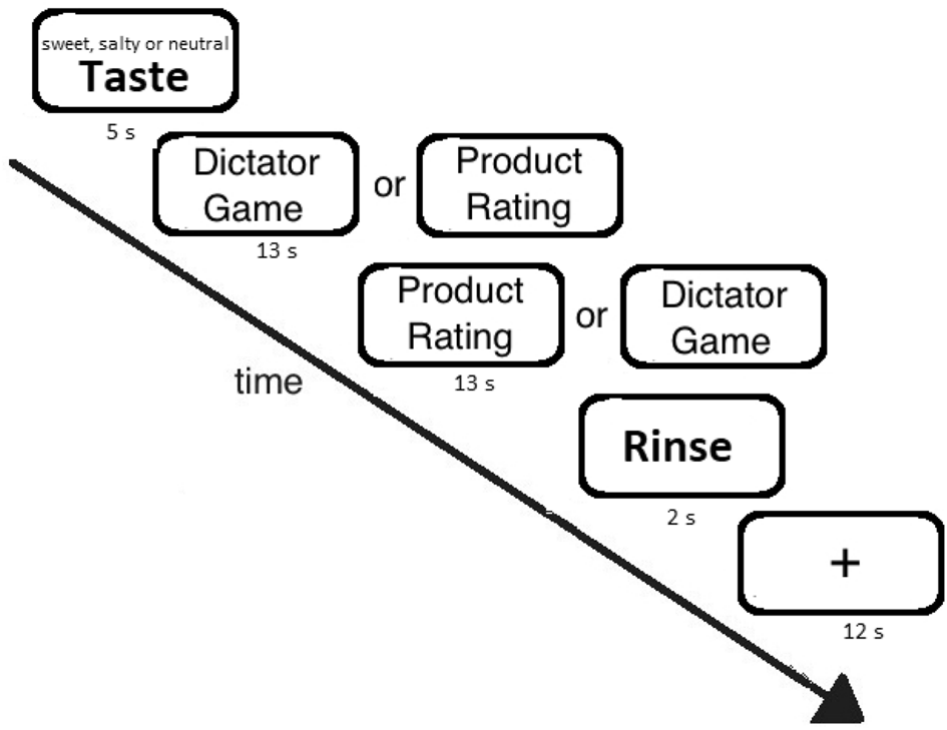
Experimental design. See text for details.

In our experimental design we examined whether the factor taste (sweet, salty, and neutral) influences social behavior in the DG. In addition, we included a control task in which participants had to rate various products. This condition was added as a second control condition to test whether taste specifically affects social behavior (or perception) or behavior in general (see Fig. 1 for the design of the study). This control task is also necessary to test whether possible activations of taste-related brain areas during the DG might be engaged simply because subjects still experience the sweet taste.

Inside the scanner orally given gustatory stimuli were delivered by a custom-build device using a tube system similar to previous studies (e.g.^37–40^). Using this gustometer participants received small amounts (about 1.5 ml) of sugary, salty, or neutral liquids. Taste stimuli were designed according to previous studies (sweet: 560 mM sucrose; salty 180 mM NaCl) (e.g.^40–42^). For rinsing and for providing a tasteless control taste we used artificial saliva (demineralized water, Natron, KaChl)^43^.

To measure social behavior participants played the DG. The DG is an economic game similar to the ultimatum game. The ultimatum game examines cooperation behavior by playing games in which an amount of money has to be distributed between two participants. Numerous studies have demonstrated that players confronted with unfair offers are willing to punish the proposer’s behavior^44,45^. In the DG the player similarly has the task to distribute an amount of money between himself and a second player, but in contrast to the ultimatum game here the responder is completely passive and has no chance to punish or react to the proposer. The first player is being told that he or she will never meet the second player and that their identity will not be revealed after the end of the study. Thus, the proposing player can act as a “dictator”, without having any worries that the second player might punish him because of the first player’s decision. Similar to the ultimatum game the DG has been examined in many studies. Since the results consistently show that individuals often forgo money in order to help others, the DG has been used to show that human behavior is not always motivated by self-interest (in contrast to rational choice models in economics)^46^.

In the current experiment we used binary DG^47,48^. In each DG the participant had to divide 15 Euro between himself and another person, who was unknown to the proposer. The player could choose one of two options of payoffs, one that favors the dictator and one that is in favor for the other player. For example, the dictator had to choose between two options. In the first (selfish) option he or she would keep 7.80 € for himself and allocates 7.20 € to the recipient. In the second (social) option he or she would keep 7.20 € for himself and give 7.80 € to the second player. The rounds of the DG differed with respect to the potential decision conflict. The proposer was told that he or she will never be going to meet the recipient and that 30% of the games would be selected at random for paying out the earned money. The monetary amount was then converted into course credits. Thus, the participants of the experiment received different credits depending on their decisions in the DG.

While lying in the scanner participants first received sweet, salty, or neutral gustatory stimuli in a random order. Participants were visually cued by a text “Taste” for 5 s. Then a new screen showed up, indicating the beginning of the DG as described above. Participants used a button box in their right hand to make their decisions. Use of right and left buttons were randomized over the different rounds of the DG. The DG lasted for 13 s. Then the participants were asked to rate a product (using a four-point Likert scale, 1 = completely undesirable, 4 = completely desirable). Various products of comparable attractiveness were shown^49^. We used this task to measure whether the effects of taste would extend to a theoretically unrelated measure (see above). After 13 s a neutral gustatory taste was given for rinsing purpose, associated with a test on screen “rinse, please swallow now”. Finally, there was a break of 12 s until the next round started.

The experiment comprised four runs (including all conditions), with a total of 60 dictator games (similar to previous studies e.g.^45,50,51^). Participants were allowed to take short breaks between the runs. The position of the tasks (DG, product rating) was pseudorandomized. Thus, half of the DGs followed the taste conditions, the other half was presented after the control task (product rating). The whole experiment lasted for about 45 min.

Visual stimuli were presented on to a screen at the end of the scanner bed close to the subject’s head. Participants viewed the visual stimuli through a mirror mounted on the receiving coil. We placed foam cushions tightly around the side of the subject’s head to minimize head motion.

### FMRI data acquisition and analysis

MRI scanning was conducted on a 3 T Siemens Tim Trio scanner (Siemens, Germany). Whole brain T2-weighted functional images were collected using echoplanar T2-weighted images (TR = 2 s, TE = 35 ms, flip angle = 80°, FOV = 224 mm, number of slices = 32, voxel size = 3.125 × 3.125 mm, slice thickness = 3.5 mm). T1-weighted structural images were acquired using an MP-RAGE sequence prior to the functional runs for anatomic reference (TR = 1650 ms, TE = 5 ms). Statistical Parametric Mapping Software (SPM12, Wellcome Department of Imaging Neuroscience, University College London, London, UK) was used to analyze the data.

Preprocessing of fMRI data included spatial realignement, coregistration, normalization into a standard anatomical space (MNI, Montreal Neurological Institute template, isotropic 3 mm voxels), and smoothing using a Gaussian kernel with an isotropic full width at half maximum (FWHM) of 8 mm. Furthermore, high pass temporal filtering with a cutoff of 128 s was used to remove low frequency drifts.

Statistical parametric maps were computed using multiple regressions with the hemodynamic response function modeled in SPM. Data analyses were performed at two levels: we first examined data on the individual subject level (fixed-effects model). Then, the resulting parameter estimates for each regressor at each voxel went into a second-level analysis (random-effects model).

In our hypothesis we assumed that sweet taste was associated with an increase of social decisions. According to the conceptual metaphor theory this effect should be based on an engagement of primary taste areas during the decision. Therefore, our analyses focused on the time window when participants made their decisions in the DG. For all trials in which participants made prosocial decisions, we calculated the following contrasts between the different taste conditions: (1) Sweet taste vs. salty taste. (2) Sweet taste vs. neutral taste. In addition, we computed the contrast salty vs. neutral taste for control reasons.

In a first step we report activity with small volume correction for a priori regions of interest (ROIs) (at p < 0.05, FWE corrected within these ROIs) for these contrasts. These ROIs were based on previous research on the taste pathway and prosocial behavior and include the rTPJ, ACC, and insula^27,52–54^. Thus, we tested whether neural activity in the rTPJ, ACC, and insula was significantly different for sweet relative to salty (and to neutral taste, respectively) during social decisions in the DG (sweet > salty taste, sweet > neutral taste).

In addition, we tested our hypothesis that brain areas engaged in primary taste processing subserve the psychological sweetness effect by using a masked analysis. Thus, we computed a mask of brain areas activated when participants actually received sweet taste (relative to neutral and salty taste, respectively) and report brain areas engaged when deciding prosocial in the DG subsequent to sweet taste experiences (relative to neutral and salty taste, respectively) that were lying in this mask.

Furthermore, we examined a ROI based on a meta-analysis on social behavior. This meta-analytic ROI was based on the term “social cognition” and included 220 studies. An additional ROI was used based on the term “monetary reward”, which included 97 studies. The ROIs were taken from the neurosynth-website (https://neurosynth.org).

In a second step we describe exploratory results on a whole brain basis. Here we considered active regions at p < 0.001 (voxel-wise threshold, uncorrected). Anatomical interpretation of the functional imaging results was performed by using the SPM anatomy toolbox.

## Results

### Behavioral results

Due to technical reasons (e.g., loss of behavioral data), we excluded three participants before subsequent analyses. Analyses of the behavioral responses during the DG showed that in 46% of the games the participants decided for prosocial allocation proposals (= giving the biggest part of money to the recipient). Hence, in about half of the total allocation games the participants showed a non-selfish, social behavior. One subject had suspicions with respect to the hypotheses of the experiment and was replaced by another participant.

**Figure 2:**
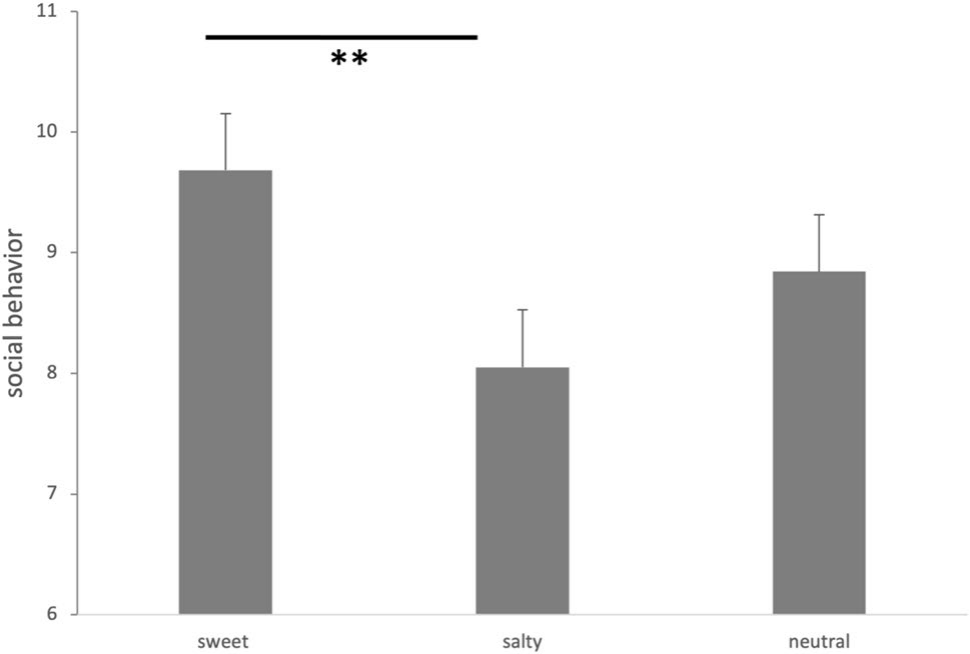
Decisions (means + standard errors) in dictator games after taste sensations. When participants experienced sweet taste before, they decided more often social (= to give the biggest part of money to someone else) relative to salty taste. Participants decided in about half of the games social (collapsed for position of the task).

To test our hypothesis that sweet taste influences later decisions to be more social, we examined whether the experience of sweet taste affected behavior in DG (number of altruistic decisions in DG, collapsed for position in the design (presented immediately after taste or after control task)) by calculating an ANOVA with the factor taste (sweet, salty, or neutral taste). We found a significant effect for taste (F (2,36) = 4.04, p = 0.03, partial eta^2^ = 0.18). Participants behaved more often socially when experiencing sweet (mean 9.68 ± 2.79) compared with salty taste (8.05 ± 3.05, t(18) = 2.92, p = 0.009; two-tailed, Cohen’s d = 0.67; see Fig. 2). In addition, sweet taste compared with neutral taste (8.84 ± 2.85) also revealed a higher frequency of social decisions but this comparison failed to reach the level of significance (t(18) = 1.35, p = 0.19, Cohen’s d = 0.31). Furthermore, comparing salty taste with neutral taste resulted in less social behavior in the subsequent DG. However, this effect was not statistically significant (t(18) = -1.46, p = 0.16, Cohen’s d = 0.33).

**Figure 3:**
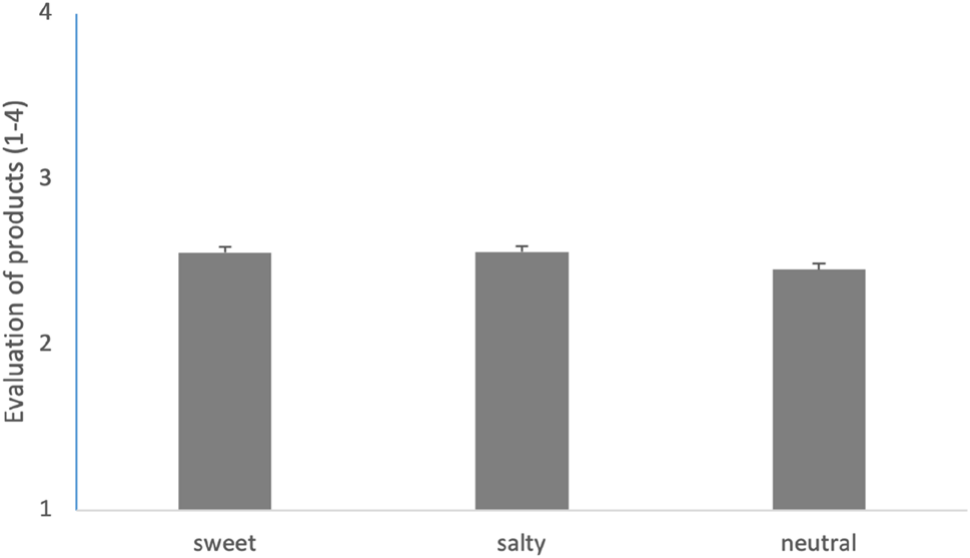
Decisions (means + standard errors) for product evaluations after taste sensations. Results were not affected by different tastes before judging the products.

Investigating analogue effects of taste for ratings of the products in the control task (collapsed over position) showed that they were not affected by taste (p > 0.10, see Fig. 3). Hence, taste seems to be associated with social behavior in the DG but does not seem to be linked to the general behavior of the participants.

### FMRI results: ROI analysis

**Figure 4:**
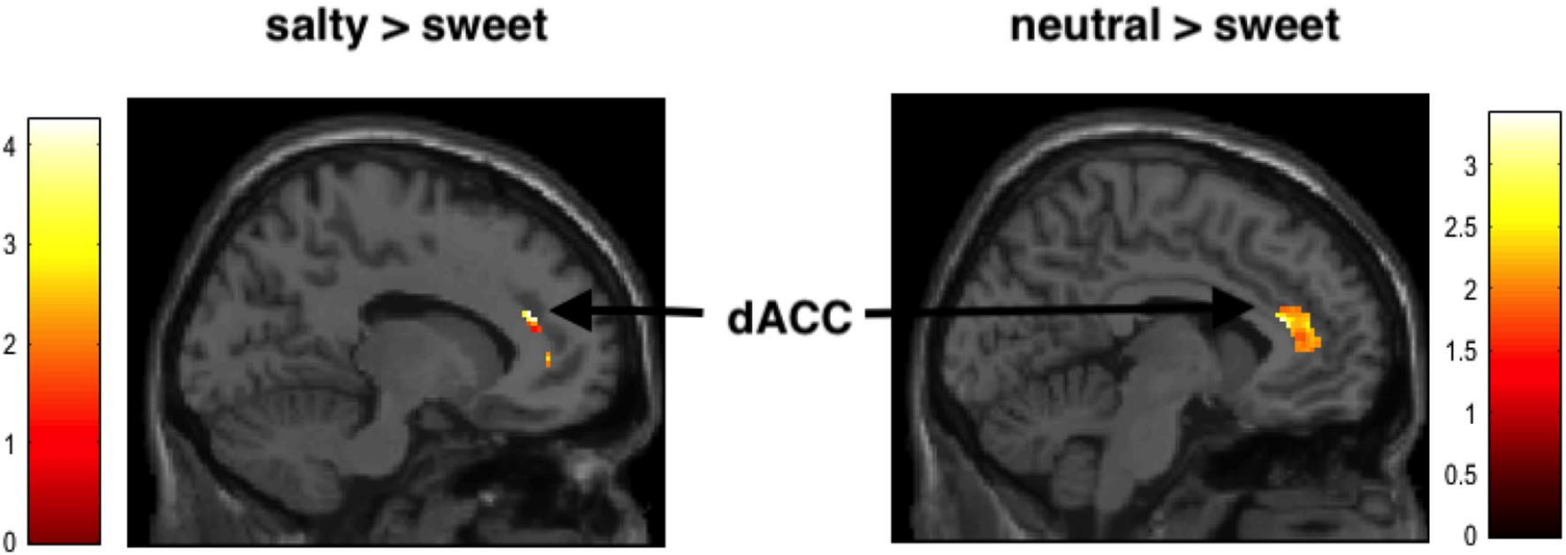
Engagement of dACC when making social decisions depending on different tastes experiences before. Results demonstrate reduced activation of the dACC compared with salty and neutral taste, respectively, when participants tasted sweet stimuli before (masked with anatomical ROI, at p < 0.05 uncorrected, for picture purpose only).

Calculating contrasts between experiencing sweet relative to salty taste before showed no significant activation (ROI analysis), even at a liberal threshold of p < 0.005 (uncorrected). In contrast, the opposite comparison salty relative to sweet taste revealed an activation of the ACC (based on ROI analysis, at p < 0.05, FWE corrected, see Table 1 and Fig. 4).

**Table 1:** Results of random effects analysis for brain responses when behaving prosocial after taste experiences (ROI analysis, at p < 0.05, corrected, FWE, L = left hemisphere, R = right hemisphere).

	Contrast	Brain region	Peak MNI location (x, y, z)	Peak z-value
Prosocial behavior	Sweet > salty taste	–	–	–
	Sweet > neutral taste	–	–	–
	Salty > neutral taste	–	–	–
	Salty > sweet taste	L dACC/SFG	−12 40 24	4.28
	Neutral > sweet taste	ACC	10 24 12	3.77
	Neutral > salty taste	R SMG	56 −44 26	3.40

Similarly, the comparison between sweet relative to neutral taste showed no active clusters, but the contrast neutral relative to sweet taste demonstrated activation of ACC (based on ROI analysis, at p < 0.05, FWE corrected). ACC signal changes of both contrasts were strongly correlated, indicating that those subjects who showed less activation of the ACC in the sweet relative to neutral task were also demonstrating less activation in this brain region when examining sweet relative to salty taste experiences (Pearson, r = 0.40, p = 0.09, two-sided).

Comparing salty and neutral taste revealed no significant brain activation. For the opposite contrast (neutral > salty) we found activity in the right supramarginal gyrus (SMG) (ROI analysis, at p < 0.05, FWE corrected).

Analyses with ROIs based on meta-analyses for neural activity related to the term “social cognition” or “monetary reward” revealed no significant activation (at p > 0.05, FWE corrected; see supplementary material S1 for further results).

FMRI results during the actual experience of sweet and salty taste showed activations in pre- and postcentral gyrus, dACC, angular gyrus, putamen, insula, and other brain areas, as expected. To test our hypothesis that brain areas of the taste pathway are engaged when participants decided prosocial in the DG subsequent sweet taste experiences, we computed a mask of brain activations when participants actually tasted the sweet stimuli. Brain activation during the DG using this mask of actual experience of sweet vs. neutral taste (or relative to salty taste, respectively) failed to surpass the level of significance (at p < 0.05, FWE corrected; see supplementary material S2 for further results).

When examining brain activations for selfish decisions during the DG results revealed no active clusters with respect to the different taste experiences before (ROI analysis, at p < 0.05 corrected).

### FMRI results: exploratory whole brain analysis

When comparing brain responses for social relative to selfish decisions in general during the DG (irrespective of taste) showed active clusters including the right TPJ/STG/posterior STS, anterior insula, right superior frontal gyrus and other brain regions (social > selfish decisions, at p < 0.001, uncorrected, whole brain analysis).

Further exploratory whole brain analysis when calculating contrasts between experiencing sweet relative to salty taste before showed no significant activation (whole brain analysis), even at a liberal threshold of p < 0.005 (uncorrected). In contrast, the opposite comparison salty relative to sweet taste revealed an activation of the ACC, angular gyrus, and other brain regions (at p < 0.001, uncorrected, see Table 2).

**Table 2:** Results of random effects analysis for brain responses when behaving prosocial after taste experiences (whole brain analysis, at p < 0.001, uncorrected, L = left hemisphere, R = right hemisphere).

	Contrast	Brain region	Peak MNI location (x, y, z)	Peak z-value
Prosocial behavior	Sweet > salty taste	–	–	–
	Sweet > neutral taste	–	–	–
	Salty > neutral taste	L cerebellum	−20, −64, −28	4.10
		L cuneus	−4, −88, 14	3.83
		L calcarine cortex	−8, −76, −4	3.66
		Brain stem	−6, −34, −34	3.58
		R lingual gyrus	12, −80, −16	3.56
	Salty > sweet taste	L dACC/SFG	−12, 40, 24	4.28
		R SFG	6, 26, 44	3.96
		L cerebellum	−16, −82, −28	3.74
		R ang. gyrus	30, −72, 40	3.61
		L cuneus	−14, −80, 32	3.46
	Neutral > sweet taste	L occipital gyrus	−44, −80, 8	4.05
		R parahippoc./hippoc	18, −20, −24	3.78
		ACC	10, 24, 12	3.77
		R ventral DC	20, −12 ,−12	3.72
	Neutral > salty taste	R SMG	56, −44, 26	3.40

Whole brain analysis of the comparison between sweet relative to neutral taste showed no active clusters, but the contrast neutral relative to sweet taste demonstrated activation of ACC, parahippocampal and hippocampal regions (at p < 0.001, uncorrected).

When comparing salty and neutral taste we found brain activation only for the occipital lobe. The opposite contrast (neutral > salty) demonstrated activity in the right SMG (at p < 0.001, uncorrected, no further brain activations).

Last, we tested brain activations for selfish decisions during the DG. Results revealed no active clusters with respect to the different taste experiences before, except for the comparison between salty and neutral taste that showed brain activation in left parietal lobe/left angular gyrus (at p < 0.001, uncorrected).

## Discussion

The current study examines the neural underpinnings of the effect of sweet taste on social intentions and behavior. Our results successfully replicated the sweet effect in the fMRI, demonstrating that sweet taste was associated with an increase in social behavior. FMRI results suggested that this effect was accompanied by a reduced engagement of the dACC.

### Neural underpinnings of the sweet effect

Several studies have demonstrated behavioral effects of sweet taste, showing effects of sweet taste on romantic feelings and approach behavior, and vice versa (e.g.^9,10^). Furthermore, effects of sweet taste on social intentions and behavior have been reported (e.g.^17^). The present study replicates these findings for the first time in a neuroimaging experiment. Our results demonstrate that briefly experiencing sweet taste made subsequent decisions more often social (compared with salty tastes).

What are the neural underpinnings of this effect of sweet taste on social behavior? FMRI results revealed a reduced engagement of the dACC associated with the psychological sweet effect (when participants decided prosocial and received sweet taste before relative to salty or neutral taste). The ACC is well-known to play a role as a central hub to integrate emotional brain areas (limbic system) with regions linked to cognitive control (prefrontal cortex), therefore it is important for a variety of functions including affect regulation, performance monitoring, error detection and general decision making^55–59^. For example, it has been demonstrated that lesions in the ACC result in impaired conflict adaption in emotional tasks, suggesting that the ACC is essential for this task^60^. More in detail, the dorsal part of the ACC has been linked to economic choice and self-control^61,62^. For example, neurons in dACC in macaques have been shown to reflect decisional dynamics for binary economic choices^63^. Remarkably, it has been shown that altruistic action in an economic task is associated with the orbitofrontal cortex and the rTPJ-STS region, whereas selfish behavior is indexed by the dACC^27^. This is in line with our findings, which showed reduced activity of the dACC when tasting something sweet relative to salty and neutral tastes and then acting altruistic.

The dictator game we have employed to measure social behavior can be described as an (emotional) conflict between keeping the biggest part of the money or giving it to the other player. We argue that the reduced activation of the dACC points to a reduction of monitoring the conflict of the economic decision, which then resulted in a higher probability to behave prosocial. This interpretation is also in line with research demonstrating a role of the ACC in attention^64,65^. For example, a recent study shows that ablation of the ACC results in reduced affective vigor and vigilance^66^.

### Why is sweet taste associated with social behavior?

Why did the experience of sweet taste result in increased helping behavior? Given that in the English language sweetness is often used to describe a social and helpful individual (“being a sweetie”), the conceptual metaphor theory has been used to explain the sweet effect on helping behavior. But this metaphor is not known in the German language, so alternative explanations should also be taken into account. For example, feeding during infancy may be important, since breast/formula milk tastes sweet. These experiences may have been consolidated (or embodied) in scaffolds of our mind and might still affect our present thinking and behavior^16^. However, our results do not show any activation of medial temporal lobe structures that might point to the involvement of memory related processes.

In addition, as mentioned above, our preference for sweet taste might be explained from an evolutionary point of view, because sweet taste signals important information with respect to plants with available glucose^5,6^. Thus, sweet taste may induce approach behavior, in contrast to avoiding behavior associated with other tastes. Numerous studies have demonstrated such links between sweet taste and approach behavior (see above, e.g.^9^). Prosocial or altruistic behavior might be understood as an example of approach behavior. In this line of explanation, sweet taste may be embodied and sometimes unconsciously guide our social behavior. In contrast to the conceptual metaphor theory, the last two explanations refer to embodiment, but not to language.

According to the embodiment theory we hypothesized that activating the sweetness effect should elicit source-domain activity in primary taste processing areas (e.g., the insula)^32^. Our results demonstrate that the dACC is involved in the sweetness effect. This brain area was engaged during actual sweet taste experience in our study and is also frequently reported in taste studies (e.g., for food preferences^67^), although it does not represent the primary gustatory cortex. However, there are pathways of the ACC to the insula^68^. Recent work revealed that the anterior insula and ACC have a close functional relationship^69^. Sweet taste may have affected the ACC via these pathways and thereby initiate the sweet taste effect on social behavior. Moreover, it has been reported that there are cingulate-amygdala pathways, which seems to play an important role in helping behavior^70^. Thus, we argue that our imaging results might provide support for the theoretical assumption that the sweetness effect engages source-domain activity in taste processing, suggesting that the sweet taste may be indeed embodied. However, since the neural representation of taste is complex and includes a network of different brain areas^31^, further studies are necessary to test this theory and to understand the mechanisms underlying the sweetness effect.

We also found that salty taste resulted in less social decisions compared with tasting neutral liquids before (although this difference failed to reach the level of significance). Those results were supported by fMRI activations, which demonstrated reduced engagement of the right SMG region when participants experienced salty taste before. This brain region has been shown to be crucial when participants try to overcome emotional egocentricity bias in social judgments tasks^71^. Hence, the salty taste seems to have an opposite effect compared to the sweet stimuli. Previous behavioral studies may not have reported these findings because they might have used less salty stimuli (e.g., crackers), whereas our study used simply salty liquids, which may have produced stronger taste experiences. Furthermore, some experimental designs may not have enough power to examine this difference (or did not include a neutral taste condition allowing to compare salty effects relative to neutral taste). Only few studies examined the effects of salty taste on behavior and thinking^9^. Future studies need to further address the psychological effects of this taste type to test whether this finding is reliable.

### Limitations

In contrast to the behavioral studies discussed above, we here show the effect in a neuroimaging environment, which required a change of the design to a more controlled lab experiment, with repeated measurements and within-subjects design. On the one hand, this adaptation of the setup to a lab experiment may have increased the power of the effect. On the other hand, the very specific setup using tubes to deliver gustatory stimuli inside the artificial environment of the scanner might also bear the risk of not replicating the effect that has been so far only reported in behavioral studies. However, although the absolute differences are small, our experiment replicated the effect and demonstrated medium effects sizes of the influence of sweet taste on behavior. In the light of recent failures to replicate behaviorally reported findings (e.g.^72^), one can speculate that for this kind of studies it may be crucial to establish a situation, where individuals are forced to decide from the gut. This seems to happen when participants had to decide immediately because someone surprisingly asks for help^17^, but also when lying inside the scanner and having only 13 s to decide between two options.

Can our results on social behavior be explained by other variables than taste? It might be objected that sweet taste may have put our participants in a general positive mood, irrespective of the social behavior task. We think that his seems unlikely, because we did not find any effects of taste on the evaluation of control pictures. Furthermore, previous behavioral studies have demonstrated that attractiveness of the sweet stimuli (or thankfulness when receiving a favorite snack^23^) does not explain the sweetness effects (e.g.^11^).

Further points that may limit our findings need to be mentioned. First, we measured prosocial behavior by the dictator game, which is only one way to measure social behavior. Future studies should replicate the results using different social tasks. Second, further control variables should be taken into account to rule out that the effect of sweet taste on helping behavior is a general effect rather than specific for social acting. Third, further tastes such as sour and bitter could be tested to determine whether these tastes have any effects on our social perception and behavior. Fourth, the impact of culture should be considered more directly in future studies, which may help to better understand the origin of the sweetness effect. Fifth, the results are based on a small sample size and need to be replicated in a larger independent sample. Previous discussion on failed replications in psychological experiments have drawn the attention to adequate sample sizes. On the one hand, it is difficult for fMRI approaches to include sample sizes comparable to behavioral studies. On the other hand, study designs in fMRI experiments often include within-subjects design and numerous repetitions, which needs to be taken into account when comparing sample sizes of imaging with behavioral studies. However, although the present study used sample sizes similar to other fMRI studies on conceptual metaphor theory and embodiment (e.g.^36^), the results have to be interpreted with caution. Last, we did not ask the participants about their food preferences. Although previous work has shown that the personal preference for sweets did not affect the psychological sweet effect^11^, future studies should control this variable.

## Conclusions

What are the implications of this research? Given that the behavioral effects may be small, practical consequences should be discussed carefully. However, it seems remarkable that alterations in ACC have been consistently reported in patients with major depression (e.g.^73^). Furthermore, it has been reported that patients with depression have an altered perception of sweet taste^74^, which might also contribute to the known reciprocal relationship between depression and obesity^75^. Given that the effects of sweet taste address prosocial and outgoing behavior, one might speculate that a training to improve sweet taste might change depressive symptoms (and perhaps also affect obesity).

Taken together, the present study demonstrates that sweet taste was associated with an increase in prosocial behavior. Our results suggest that this sweet taste effect is related to less activation of the dACC, pointing to a decreased selfish behavior when experiencing sweet taste beforehand^27^. The results provide a first step to understand the neural basis of the sweetness effect. We conclude that sweet taste has remarkable and versatile effects on our perception, thinking, and behavior.

## Supplementary Information

Supplementary Information.

## Data Availability

The data presented in this study are available on request from the corresponding author.
