# Supplementary material for: Experiencing sweet taste is associated with an increase in prosocial behavior
Source: Sci Rep. 2023 Feb 2;13:1954. doi: 10.1038/s41598-023-28553-9 (PMC9894851; doi:10.1038/s41598-023-28553-9)
Supplement: Supplementary file 1 — Supplementary Information. [file 41598_2023_28553_MOESM1_ESM.docx]

# Supplementary Material

**Experiencing sweet taste is associated with an increase in prosocial behavior**

Michael Schaefer ^1^ *, Anja Kühnel ^1^, Felix Schweitzer ^1^, Franziska Rumpel ^1^, Matti Gärtner ^1^

**^1^** Medical School Berlin, 12247 Berlin, Germany

**^2^** Otto-von-Guericke Business School Magdeburg, 39106 Magdeburg, Germany

S1: Further results of meta-analytic ROIs

S2: Further results of masked analysis

**S1:** Further results of meta-analytic ROIs

Using a meta-analytic ROI for „social cognition” with a more liberal threshold revealed activation of the right middle temporal gyrus (46 -46 4, at p < 0.005, uncorrected) for the contrast salty > neutral. For the „monetary reward” network a liberal threshold showed activity in the right putamen (26 10 -2) for salty > sweet. There were no other significant brain activations.

|  |  |
| --- | --- |

**S2:** Further results of masked analysis

When using a more liberal threshold the masked analysis resulted in an activation of dACC for both contrasts (salty taste > sweet taste, -12, 24, 30, z = 3.49; neutral taste > sweet taste, -16, 36, 24, z = 3.26, at p < 0.005, uncorrected). This masked analysis revealed no other significant brain activations. Comparing salty and neutral taste (and vice versa) during the DG with a mask of actual experienced salty taste (relative to neutral taste) showed no significant brain activation.
